# Complete Occlusion of Anterior Capsular Opening in Patient Operated for Cataract With Penetrating Keratoplasty

**DOI:** 10.7759/cureus.25178

**Published:** 2022-05-21

**Authors:** Rajesh S Joshi, Preeti Wadekar

**Affiliations:** 1 Ophthalmology, Government Medical College, Nagpur, IND; 2 Ophthalmology, Government Medical College, Akola, IND

**Keywords:** cataract surgery, phacoemusification, neodymium-doped yttrium aluminum garnet laser, capsulotomy, ccc, intraocular lens material, anterior capsular contraction, intraocular lens decentration, anterior capsular fibrosis

## Abstract

A 68-year-old female underwent a full-thickness penetrating keratoplasty (PK) and developed a mature cataract for which she was operated on using the phacoemulsification technique with the implantation of polymethyl methacrylate lens. The patient developed diminished vision one month after the cataract surgery. The patient had a contraction of the anterior capsular opening. Neodymium-doped yttrium aluminum garnet laser (ND:YAG) anterior capsulotomy was performed to create an opening in the anterior capsule, following which the patient regained her vision. To the best of our knowledge, this is the first report of early anterior capsular contraction in a patient operated for PK.

## Introduction

A continuous curvilinear capsulotomy (CCC) lets surgeons safely perform phacoemulsification and implantation of a posterior chamber intraocular lens (IOL) in the bag. However, it carries the risk of anterior capsular contraction (ACC). ACC has been reported in cataract surgery patients with pseudoexfoliation, high myopia, uveitis, and retinitis pigmentosa [1-4]. Diabetes, myotonic dystrophy, and a systemic tendency of hypertrophic scarring have been implicated as risk factors for the development of ACC [5-7]. ACC can diminish vision if it obscures the visual axis or the IOL becomes decentered. We report a case of early ACC in a patient who underwent cataract corrective surgery using the phacoemulsification technique and initially underwent full-thickness penetrating keratoplasty (PK) for corneal opacity.

## Case presentation

A 68-year-old female presented four weeks after cataract surgery with diminished vision (20/200) in the right eye. Slit-lamp examination revealed complete closure of the anterior capsular opening by a thick fibrotic membrane (Video 1).

**Video 1:** Showing phimosis of the capsulorhexis opening. A thick fibrotic membrane is seen in the center.

**Figure 1:**
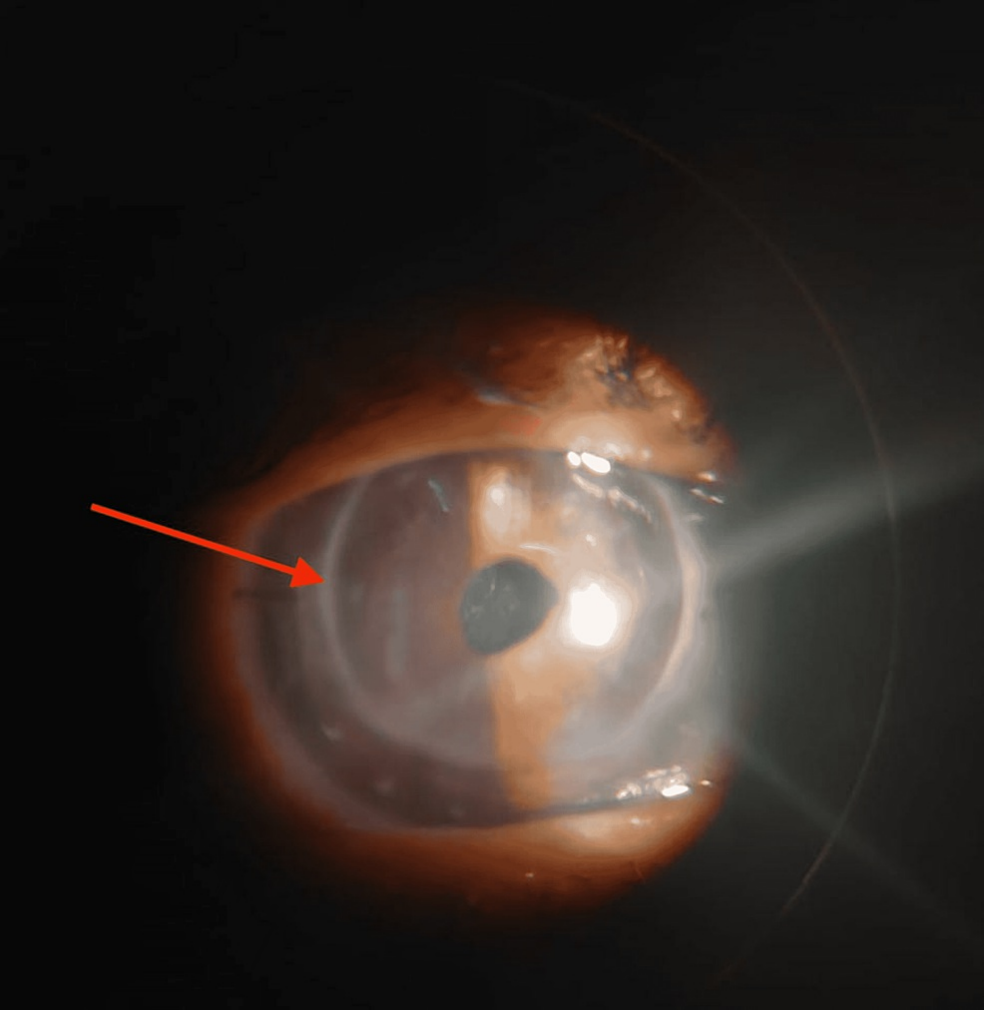
Showing post ND:YAG laser anterior capsulotomy opening. A clear central area of the pupil is seen. The arrow shows graft host junction.

Her intraocular pressure by applanation tonometer was 14 mmHg. Retinal examination by ophthalmoscopy showed a red glow only. The patient had previously received an uneventful, uncomplicated temporal clear corneal phacoemulsification with implantation of a polymethyl methacrylate IOL one month earlier. The diameter of the capsulorhexis was 5.5 mm. Vacuum polishing of the anterior capsular rim and the equatorial area had been performed. We noted no zonular weakness during any of the surgical steps. Two weeks postoperatively, the patient achieved an unaided visual acuity of 20/80, and clinical examination of the anterior segment on slit-lamp biomicroscopy revealed no remarkable findings. She had no history of uveitis, pseudoexfoliation, or high myopia. She had no history of diabetes or systemic scarring. The contralateral eye had a visual acuity of 20/100 and had central macular grade corneal opacity. Neodymium-doped yttrium aluminum garnet (ND:YAG) laser radial anterior capsulotomy was performed (total energy: 44 mJ) for the occluded capsulorhexis opening, and the patient regained 20/40 vision at the third postoperative visit (Figure 1).

## Discussion

ACC is a complication associated with CCC. Complete occlusion of the capsulorhexis opening is a severe form of ACC. Three major factors associated with ACC are the size of the CCC, IOL material, and preexisting conditions. A small CCC (i.e., 5 to 5.5 mm) has a higher risk of ACC than a large CCC (i.e., >5.5 mm) [8]. The IOL biomaterial also plays an important role in the development of ACC, with plate haptic silicone lenses reportedly associated with a higher risk of ACC than acrylic lenses [9]. However, ACC may be seen in all types of IOLs. Hydrophilic IOL demonstrates a higher incidence of ACC than the hydrophobic IOLs because the hydrophobic surface prevents the attachment of the lens epithelial surface to the IOL, reducing the development of ACC. Firm attachment of the CCC margin on the IOL optic may prevent the development of ACC. 

Ocular risk factors implicated in the development of ACC include pseudoexfoliation syndrome, uveitis, high myopia, retinitis pigmentosa, and zonular weakness, whereas systemic risk factors include diabetes, myotonic dystrophy, and a tendency for hypertrophic scarring. None of the mentioned risk factors were present in our case. Patient vision may be impaired due to the opacification of the media or tilting and decentration of the IOL. Our patient’s diminished vision was due to the presence of a fibrotic membrane in the visual axis. 

The pathogenic mechanism causing the development of ACC is unknown; however, ACC may be due to the proliferation of residual anterior lens epithelial cells that lead to fibrous metaplasia and the occlusion of the capsulorhexis opening by the fibrous tissue membrane [10]. In our case, we performed vacuum polishing of the equatorial area of the capsular bag and the rim of the anterior capsule. 

Another proposed mechanism of ACC is the reaction to the residual lens material. The ACC in the present case could not have been caused due to residual lens matter because a thorough cortical clean-up was performed, and the initial follow-up examination revealed no cortical matter. However, as our patient underwent full-thickness PK, the possibility of zonular weakness being the cause of the ACC in our case cannot be denied. However, we saw no signs of subluxation of the lens preoperatively and intraoperatively. 

The development of uveitis postoperatively, triggered by the cataract surgery, could cause ACC in our patient. She was on a once-daily local prednisolone acetate eye drops before the cataract surgery and until the last follow-up examination. 

ACC can be surgically treated or by ND:YAG laser, as done in this case. Early intervention using an ND:YAG in cases with ACC has been reported as safe [10]. Our patient received early intervention because her contralateral eye had a poor vision due to corneal opacity. As of her six-month follow-up, we found no complications in our patient after ND:YAG capsulotomy. 

## Conclusions

As our case report suggests, severe ACC can develop in a patient with a history of PK who received cataract corrective surgery with a polymethyl methacrylate lens implantation. Early ND:YAG anterior capsulotomy relieves contraction and is a safe and effective option to restore vision. To the best of our knowledge, this is the first report describing ACC in a patient with a history of PK.
